# New frontiers in healthcare environmental hygiene: thoughts from the 2022 healthcare cleaning forum

**DOI:** 10.1186/s13756-022-01185-w

**Published:** 2023-02-07

**Authors:** Alexandra Peters, Pierre Parneix, Martin Kiernan, Juliëtte A. Severin, Tracey Gauci, Didier Pittet

**Affiliations:** 1 Infection Control Programme and WHO Collaborating Center On Infection Prevention and Control and Antimicrobial Resistance, Hospitals and Faculty of Medicine, University of Geneva, Geneva, Switzerland; 2 Nouvelle Aquitaine Health Care-Associated Infection Control Centre, Bordeaux University Hospital, Bordeaux, France; 3 Richard Wells Research Centre, University of West London, London, UK; 4 Department of Medical Microbiology and Infectious Diseases, Erasmus MC University Medical Centre Rotterdam, Rotterdam, The Netherlands; 5 Hywel Dda University Health Board, NHS Wales, Carmarthen, UK

**Keywords:** Infection prevention and control, Environmental hygiene, Cleaning, Sustainability

## Abstract

Healthcare environmental hygiene (HEH) has become recognized as being increasingly important for patient safety and the prevention of healthcare-associated infections. At the 2022 Healthcare Cleaning Forum at Interclean in Amsterdam, the academic lectures focused on a series of main areas of interest. These areas are indicative of some of the main trends and avenues for research in the coming years. Both industry and academia need to take steps to continue the momentum of HEH as we transition out of the acute phase of the Covid-19 pandemic. There is a need for new ways to facilitate collaboration between the academic and private sectors. The Clean Hospitals® network was presented in the context of the need for both cross-disciplinarity and evidence-based interventions in HEH. Governmental bodies have also become more involved in the field, and both the German DIN 13603 standard and the UK NHS Cleaning Standards were analyzed and compared. The challenge of environmental pathogens was explored through the example of how *P. aeruginosa* persists in the healthcare environment. New innovations in HEH were presented, from digitalization to tracking, and automated disinfection to antimicrobial surfaces. The need for sustainability in HEH was also explored, focusing on the burden of waste, the need for a circular economy, and trends towards increasingly local provision of goods and services. The continued focus on and expansion of these areas of HEH will result in safer patient care and contribute to better health systems.

## Introduction

The last few years have been monumental for healthcare environmental hygiene (HEH), partly due to the Covid-19 pandemic, but not exclusively. The scope of HEH includes surface cleaning and disinfection, air control, water control, waste management, sterilization and device processing and laundry. Since 2015, there has been an exponential growth in good quality studies that tie improvements in HEH to a reduction in healthcare-associated infections [1]. The 2022 Healthcare Cleaning Forum at Interclean in Amsterdam had a very different atmosphere than the last edition in 2018. There was no more need to convince anyone about the importance of environmental cleaning in healthcare- the last few years of the pandemic had thrust HEH into the spotlight. This trend was reflected in the show participation; around 25% of the visitors to Interclean had come to see the Healthcare Cleaning Forum which was organized in cooperation with Clean Hospitals.

The field of HEH is beginning to come into its own, and the content of the lectures and presentations at the Healthcare Cleaning Forum reflected this. They looked at the impact of Covid-19, and how to keep momentum in the field moving forward, development of new international guidance in HEH, the need for and role of evidence-based interventions, case studies for the role of design and the built environment in combatting environmental pathogens, and urgent calls for innovation and sustainability in the field.

Covid-19 sensitized the whole world to contact transmission. In the early stages of the pandemic, facilities operated in a setting of knowledge gaps, uncertainty, fear, and a lack of supplies. Later, a fatigued workforce and a market flooded with products of varying efficacy would prove to be new challenges for HEH. The disease also spread in unexpected ways; although it was expected that patients would be the cause of nosocomial spread, Covid-19 was often spread through the population of healthcare workers sharing lunch or carpooling to their patients [2].

### The Clean Hospitals approach

The Clean Hospitals initiative, launched at the 2018 Healthcare Cleaning Forum, has grown into a collaborative public private partnership with the common goals for increased communication, improved products and practices and better patient safety. The network proved useful during the pandemic, as industry and academic partners were kept abreast of the newest challenges in the field.

The Clean Hospitals academic taskforce worked on two main pieces of research. The first was a systematic review to assess the impact of interventions in the healthcare environment on patient outcomes, wither healthcare-associated infections (HAIs) or patient colonization [1]. Eighty-eight percent of included studies showed some kind of reduction in colonization or HAI for at least one of the microorganisms tested, and 58% showed a significant reduction in all of the microorganisms tested. The studies identified make up the growing body of work that demonstrates the key importance of environmental hygiene to patient safety.

The second project, which is still ongoing, is the development of a tool for facilities to assess how well their HEH programs work, and give indications of how to improve them. The Healthcare Environmental Hygiene Self-Assessment Framework was first tested as an international pilot survey [3], and is currently undergoing the final stage of development before its projected dissemination in 2023.

### New guidance

During Covid-19, new guidelines and expert reviews were developed around the world, and healthcare facilities became increasingly aware of the role of the environment in the transmission of HAIs. In 2021, two new sets of national guidance for cleaning were launched in and around Europe. In Germany, the German Standards Institute (DIN) 13603 standard was the first national guidance produced; it applies to all healthcare providers [4]. In England, the National Health Service (NHS) Cleaning Standards launched a second and revised version that did not apply to the NHS in Wales, Scotland or Northern Ireland or to independent healthcare providers [5]. Although independently produced, there were some similarities and some differences in the approaches (Table 1). Both sets had multiple inputs from stakeholders’ groups. However in England, professional societies like the Infection Prevention Society and the Healthcare Infection Society did not endorse the guidance, whereas in Germany, the Robert Koch Institute was instrumental in the creation process.

**Table 1:** Comparison of key aspects of England's NHS and Germany's DIN guidelines

	NHS (England) cleaning standards	Germany DIN 13063
Audit and inspection method	Visual only; subjective. Equipment and items cleaned by the clinical staff are part of audit	Comprehensive description of methods including visual, ATP, Fluorescent marking, microbiological. Clinical items not explicitly included
Audit results delivery	Star rating visually displayed in each area. Star ratings vary by a complex functional risk system	Results need to be communicated to the employees at execution level in an understandable and practical form and left to local delivery method
Evidence base	No literature review; recommendations not supported by evidence in the scientific literature	No literature review; recommendations supported by evidence in the scientific literature
Guidance applicable to	NHS in England only, not Wales, Scotland or independent healthcare providers	All healthcare providers in Germany
Involvement of specialists	Professional cleaning organisations and public institutions; not endorsed by nursing or infection prevention-related professional societies	Collaborative between professional infection prevention-related societies, public institutions, companies in the cleaning industry, their suppliers and associations
Outline of responsibilities	Role of professional cleaners is described. Clear statements about the role of clinical staff in decontamination of clinical equipment	Comprehensive; client/service recipient defines interfaces for defining a separation of devices
Training needs	Mentioned but little detail provided	Comprehensive; outlines detail on training, requirement for expertise and stating that the content of training should be monitored
Colour coding used to separate different areas	Yes	Yes
Use of ‘novel’ technologies such as UV, Gaseous decontamination	Not mentioned	Not mentioned

The NHS standards provide clear advice and guidance on what cleaning is required, and how organizations can demonstrate cleaning services meet these standards. Although they state that recommendations are based on sound evidence and accepted good practices, no literature review was ever conducted and there is no evidence of a systematic process.

One positive development is that when addressing recommendations for cleaning, the NHS standards make clear statements about the role of clinical staff in environmental decontamination. The DIN standard however does not define clear responsibilities for staff groups, instead leaving this for individual organizations to define. It does however describe clear methodologies for cleaning and disinfecting a variety of surfaces. Both standards mention the need for training, however the DIN is far more prescriptive in describing necessary content and levels of attainment. Audit is also covered in both standards however methodologies differ: where the NHS system is a subjective qualitative visual assessment, whilst the DIN gives great detail on quantitative assessment of effectiveness of cleaning.

The largest change in the second version of the NHS Standard is the adoption of a collaborative approach for the responsibility for cleaning, and so combined working is necessary to achieve the stated audit standards, which are then displayed as a ‘star’ rating. The effect of this on public confidence has not been measured to date. There is no such approach in the DIN standard, which adopts an accepted approach to the determination of quality, using the three interdependent aspects of structure, process and outcome. Interestingly, although both sets of standards describe cleaning and decontamination processes in varying degrees of detail, neither has taken the opportunity to include accepted and well-established evidence-bases automated technologies such as gaseous hydrogen peroxide and ultraviolet light technologies.

Both sets of standards are a step forward for their countries, the NHS including the clinical staff in responsibilities for cleaning and the DIN for being the first national guidance. Both however would benefit from a more transparent description of the evidence base and strength of evidence from which the recommendations have been derived.

### Case study of an environmental pathogen: Pseudomonas aeruginosa

A safe hospital is a clean hospital, but what exactly “clean” means can vary. In the context of preventing HAIs, it could be interpreted as a microbiologically safe hospital without pathogenic microorganisms in its environment. *P. aeruginosa* is especially capable of surviving in the hospital environment, with sinks as the most frequently reported reservoir [6–9]. *P. aeruginosa* bacteria form biofilms in the lumen of pipelines, from which cells may be released during sink use and spread outside of drains within droplets or as aerosols. Surfaces in the patient environment may become contaminated, and ultimately reach the patient. A recent analysis showed that the vast majority of *P. aeruginosa* infections (86.3%) were transmitted through the environment as opposed to cross-transmission from other individuals or patients. Bacterial drain reservoirs are notoriously difficult to eradicate, as commonly-used hospital disinfectants do not remove biofilms, and recolonization may occur after exposure to contaminated materials or retrograde growth from p-traps.

When it is not feasible to remove such pathogenic bacteria, elimination of the complete reservoir can be considered; an intervention which several hospitals have implemented [10–12]. The removal of sinks was performed in the framework of a bundle focused on water-free patient care. Though this intervention had an effect in all three studies, the removal of sinks is not feasible outside of intensive care units, and shower drains may be reservoirs for *P. aeruginosa* as well [13].

If neither elimination of the pathogen, nor the complete removal of the reservoir is possible, elimination or control of the transmission route of pathogenic microorganisms from or via the environment is a pragmatic approach. A range of such interventions have been published, often focused on an improved sink design [6, 14, 15]. Most of these interventions showed significant reductions in transmission, though control was not fully achieved.

To identify alternative solutions, a better understanding of the hospital’s microbiota and the environmental biofilms is key. Though recent studies using (meta)genomic analyses of the hospital environment have provided some insights into the hospital microbiota, they did not provide support for the development of new interventions such as probiotic-based treatments [16–18]. An analysis using a culture-based approach based on MALDI-TOF mass spectrometry of sink biofilms did provide some modest leads, but additional experiments are needed to draw more firm conclusions on which microorganisms enable or inhibit *P. aeruginosa* persistence [8]. Enzymatic, probiotic [18] or phage-based approaches should also be explored. Innovative approaches are needed to address persistent environmental reservoirs of bacteria and help create microbiologically safe hospitals. The question is how to best foster these types of approaches.

### Innovation in environmental hygiene

Innovation is everywhere in our daily-life including the healthcare system. Infection control and environmental hygiene have been expected to take advantage of these progresses on the condition that we are able to assess the efficacy and the impact of new available technologies [19].

Electronic health records in hospitals and the software to instantaneously aggregate them, provide important data that should be available to infection control teams. If collected and analyzed appropriately, this data can provide practitioners knowledge which can, in turn, be transformed into action, and potentially improve infection control [20].

Other types of digitalization and tracking are now becoming more common in environmental cleaning and disinfection. Modern technology can provide autonomy to the professional, while performing continuous monitoring, enable early detection of hygiene failures, and facilitate quick interventions. This can ultimately prevent outbreaks and mitigate both the human and financial costs associated with HAIs. Managers should encourage these innovations and their use in a fair manner, by focusing on quality improvement and not on individuals blame for failures.

It is desirable to technology to automatize important tasks that are difficult for humans to perform consistently. This is especially the case for robots that are quickly becoming ubiquitous in the field of cleaning and disinfection. Ultra violet-C (UVC) disinfection, for example, is a promising technology with demonstrated efficacy. However, international quality standards are lacking, and are important in order for consumers to fully rely on such technology where appropriate. Such technologies have additional effects beyond their efficacy- implementing visible technologies has been shown to improve safety climates in hospitals by increasing the confidence of both patients and healthcare workers [21].

Antimicrobial surfaces are based on technologies that either repel or kill microorganisms when applied to a surface [22]. The field of possible applications of such technologies is wide and still growing [23]. A French national organization for standardization/ Association Française de Normalisation (AFNOR) efficacy standard (NF S 90-700) has been published in 2019 and is the basis for what will become the ISO standard. Discussions are still underway concerning the appropriate efficacy requirements and the differentiation between similar technologies, such as for opaque versus transparent surfaces. The expected goal of antimicrobial surfaces is to help reduce the transmission burden, especially on high-touch surfaces [24].

Innovation in environmental hygiene should not be seen as an endpoint nor a magic bullet but as components of an infection control strategy that can be added in intervention bundles. Industry should focus on demonstrating the immediate efficacy of such technology and the infection control experts should decide when, where and how to use it to maximize its impact.

### Sustainability

The entirety of this research and projects and new technologies and trends need to share a common trait; they must all be sustainable to implement. Sustainability means meeting the needs of the present without compromising the needs of future generations. For healthcare facilities, there is often a tradeoff (sometimes perceived, sometimes real) of sustainability versus efficacy.

The World Meteorological Organization predicts 50/50 odds that temps will increase between 1.5 and 2C over pre-industrial levels, for one year over the next five [25]. This means that there will be irreversible and perhaps catastrophic changes to our climate. Globally, the healthcare sector is responsible for almost 5% of emissions [26]. A global analysis of health care waste in the context of Covid-19 showed hundreds of thousands tons of additional waste from Covid-19 test kits, personal protective equipment (PPE), vaccine production etc. [27, 28] Beyond the quantity of waste, the WHO estimates that 1 in 3 healthcare facilities does not safely manage its waste [27].

Countries are beginning to take steps to try to reach net zero, which means cutting emissions as close to zero as possible and reabsorb all remaining emissions into healthy oceans and forests. The UK’s target is to reach net zero by 2050 [29]. The UK’s NHS is aiming to reach net zero by 2040 [30]. While some aspects are in control of institutions, others are not. IPC is often still not wholly evidence-based, and this needs to improve so that resources are not used when it is not necessary to do so. PPE needs to be used better and more precisely. In many ways healthcare facilities need to go “back to the future” by having locally, decentralized infrastructure in order to support reuse and reprocessing where appropriate.

Developing a circular economy is central to making it sustainable. In 2020, 100 billion tons of new materials entered the world economy, and only 8.6 was circular [31]. The World Economic Forum estimates that 70% more virgin materials were extracted from the Earth than what it can safely replenish [31]. Not only do we need to use less, but we need to make things last longer, utilize renewable energy and regenerative materials, and plan how to reuse and recycle from the very beginning, instead of as an afterthought. Recycling should be the end point of a circular economy, not the first thing tried. The cost structure of materials is a major issue, as new materials are often much cheaper than recycled ones [32]. This creates frequent incompatibility between sustainability and affordability.

It is also increasingly clear that infection preventionists need to participate in the decision making around sustainability in healthcare in order to prevent new measures from having a negative effect on efficacy. If infection control experts do not get involved in this agenda, someone who is not an expert will set it. Further challenges include a lack of accountability, and social equity. It is also important to talk about responsibility, both individually and collectively at the institutional and governmental levels.

Institutions can begin to take steps in a number of ways. First, they can reduce the use of chlorine-based products [33] and adapt policies to infection prevention needs as new information evolves. It is important to reduce reliance on single use equipment unless absolutely necessary. Healthcare facilities used to reuse more, but fear during AIDS pandemic pushed facilities towards single use, even for surgical caps and gowns that could be laundered. High-cost, low-volume medical devices are often discarded simply on the advice of the manufacturer. Healthcare facilities need to work with manufacturers to change this, and to maximize the safe reusability of equipment.

## Conclusion

The future of HEH is clear as the field becomes more recognized as being key component of successful infection prevention strategy. Still, more research is needed to prove the efficacy of standard interventions and of recently developed technologies. New ways of managing the built environment need to be explored and innovation needs to be evidence-based and adopted in a context that is helpful to and respectful of the environmental services staff and our planet.

Networks such as Clean Hospitals need to continue to work on defining the research agenda globally as well as creating awareness for the field. Healthcare facilities around the world need to be able to analyze their own programs in the context of best practices, and tools must be developed to help facilitate improvement. Sustainable international norms and guidelines should be developed in order for institutions to implement universal minimum standards for quality while ensuring that future generations can enjoy the same level of access as ours.

## Data Availability

There was no data generated, but we will gladly share whatever information may be requested.

## Abbreviations

AFNOR: Association Française de Normalisation/French national organization for standardization
DIN: Deutsches Institut für Normung/German Standards Institute
HAI: Healthcare-associated infection
HEH: Healthcare environmental hygiene
NHS: National health service
PPE: Personal protective equipment
UVC: Ultra violet-C
